# Report of Unfair Discipline at School and Associations with Health Risk Behaviors and Experiences — Youth Risk Behavior Survey, United States, 2023

**DOI:** 10.15585/mmwr.su7304a8

**Published:** 2024-10-10

**Authors:** Kathleen H. Krause, Charles Bell, Bajha Jordan, Michelle Carman-McClanahan, Carmen Ashley, Izraelle I. McKinnon, Desmond Banks, Jorge V. Verlenden, Ari Fodeman, Loredona Arrey, Connie Lim, Sherry Everett Jones, Jonetta J. Mpofu

**Affiliations:** ^1^Division of Adolescent and School Health, National Center for Chronic Disease Prevention and Health Promotion, CDC, Atlanta, Georgia; ^2^Department of Criminal Justice Sciences, Illinois State University, Normal, Illinois; ^3^Behavioral, Social, and Health Education Sciences, Rollins School of Public Health, Emory University, Atlanta, Georgia; ^4^Epidemic Intelligence Service, CDC, Atlanta, Georgia; ^5^Office of Health Equity, CDC, Atlanta, Georgia; ^6^U.S. Public Health Service Commissioned Corps, Rockville, Maryland

## Abstract

Relatively little is known about the association between school discipline and student health and well-being. Using CDC’s 2023 Youth Risk Behavior Survey, CDC analyzed the prevalence of report of unfair discipline at school and associations with experiences at school, mental health, suicidal thoughts and behaviors, and health risk behaviors among high school students overall and stratified by race and ethnicity. Prevalence estimates, prevalence differences, and prevalence ratios adjusted for race (in overall models), grade, and sex were calculated. Overall, 19.3% of students reported receiving unfair discipline during the previous 12 months; Black or African American students had a higher prevalence (23.1%) compared with Hispanic or Latino students (18.4%) and White students (18.1%). Unfair discipline was reported among a majority of students who describe their sexual identity in some other way (besides gay, heterosexual, lesbian, bisexual, or questioning) for American Indian or Alaska Native (81.7%) and multiracial (57.1%) subgroups. Overall, report of unfair discipline was associated with every health risk behavior and experience examined, including being bullied at school or electronically, skipping school due to feeling unsafe, carrying a weapon at school, prescription opioid misuse, poor mental health, persistent feelings of sadness or hopelessness, seriously considered attempting suicide, and attempted suicide. This pattern of association was similar among most student groups in models stratified by race and ethnicity. This analysis is the first to demonstrate, among a nationally representative sample of high school students, that reports of unfair discipline are associated with various health risk behaviors and experiences. With these findings, public health and education practitioners can create interventions that equitably promote safe, supportive, and inclusive school environments for student health.

## Introduction

Creating safe and supportive school environments is a goal for CDC and other Federal partners, as well as the school community consisting of students, families, teachers, staff members, and school administrators. Ideally, school discipline sets boundaries that are needed to create a school climate where all students can achieve success in academics and maintain health and well-being (*1*). Schools use discipline as a tool to address behavior that interferes with student learning or could affect the safety of the school environment (*1*). However, it is widely recognized that how discipline is implemented within schools can be problematic. Although many school systems have moved to create disciplinary methods that are more restorative, school discipline still most often encompasses exclusionary discipline and less severe forms of punishment, such as being sent to the principal’s office. The Office for Civil Rights in the U.S. Department of Education defines exclusionary discipline as “the formal or informal removal, whether on a short-term or long-term basis, of a student from a class, school, or other educational program or activity for violating a school rule or code of conduct…includ[ing] detentions, in-school suspensions, out-of-school suspensions, suspensions from riding the school bus, expulsions, disciplinary transfers to alternative schools, referrals to law enforcement, and school related arrests” (*2*). Since 1990, decades of data and research have documented the negative outcomes linked to the receipt of these types of school discipline (*3*–*10*). Within the past 15 years, the American Psychological Association (*9*) and the American Academy of Pediatrics (*8*) have released reports against the broad and frequent use of exclusionary school discipline, citing the association between these types of discipline and poor academic outcomes, the link between these types of discipline and arrest and incarceration, and the ineffectiveness of these disciplinary policies in creating a safer school environment.

Few studies have investigated the relation between discipline and the health and well-being of students. A systematic review published in 2023 spanning the years 1990–2020 identified 19 studies that focused on the association between receipt of school discipline and health. Of these studies, 13 found a significant association (*10*). For example, report of suspension was associated with current tobacco use, drug use as an adult, clinical diagnosis of sexually transmitted diseases, antisocial behavior, self-injury for which the student received medical attention, depression, moderate to severe depressive symptoms, and death by suicide. In addition, report of suspension or detention was associated with future smoking experimentation. Report of suspension or expulsion was associated with borderline personality disorder and receipt of mental health services, and school-level disparities in the proportion of Black or African American (Black) students suspended compared with White students was associated with adjustment problems, such as self-report of getting mad easily (*10*). Despite this evidence, additional studies are needed for a more in-depth exploration of the link between school discipline and health.

Investigating the association between discipline and health is important to understand and promote health equity in schools. Extensive research has documented disparities among K–12 students in the receipt of discipline by demographic characteristics; male students compared with female students, students with disabilities compared with those without, and Black students compared with White students are disproportionately disciplined (*3*,*4*). The U.S. Department of Education has described the use of discipline as discriminatory against youths of color, and Black students in particular, creating disparities categorized as widespread and persistent (*11*). Since at least 1974 (*12*), schools have applied discipline disproportionately to Black students, and since the 1990s, the disproportionality for Black students has been at a rate two to three times higher relative to their enrollment and compared with White students (*13*–*15*). Broadening the extant literature to document the relation between discipline and health among Black students, to whom schools disproportionately apply discipline, is important. Regarding sexual identity, studies have also documented that students identifying as bisexual, gay, lesbian, questioning, or transgender are disciplined more often compared with heterosexual students (*4*). Identifying the health risk behaviors and experiences associated with unfair discipline among all students who experience bias, discrimination, marginalization, and racism because of their race and ethnicity, sex, sexual identity, or a combination of these characteristics is the first step needed to cultivate a school environment for students that is safe, supportive, inclusive, and fair.

This report establishes the prevalence and examines associations between the student report of unfair discipline at school, overall and stratified by race and ethnicity, and examines associations between the report of unfair discipline with health risk behaviors and experiences. Although the literature discussed previously has measured the objective receipt or report of discipline, this report focuses instead on students’ report of whether they have received discipline that they believe to be unfair. In 2023, the U.S. Department of Education released guidance for schools to cultivate safe, supportive, inclusive, and fair school climates, outlining that a key strategy is to ensure that schools implement discipline fairly (*16*). Therefore, understanding receipt of discipline reported by students to be unfair, and the association between unfair discipline and health marks a novel contribution to the literature. In addition, this report addresses two major gaps in the literature on school discipline. First, this is the first analysis to investigate the association between report of unfair discipline and various health risk behaviors and experiences among a nationally representative sample of U.S. high school students. Second, this analysis is the first to include a comprehensive examination of the relation between discipline and health among many racial and ethnic categories, not limited to Black or White student experiences, and among students of diverse sexual identities. The findings in this report will be useful for public health practitioners, school leaders, teachers and staff members, students, and their families to begin to understand the full scope of health risk behaviors and experiences associated with report of unfair discipline. With these findings, public health and education practitioners can create interventions that equitably promote safe, supportive, and inclusive school environments for student health.

## Methods

### Data Source

This report includes data from the 2023 YRBS (N = 20,103), a cross-sectional, school-based survey conducted biennially since 1991. Each survey year, CDC collects data from a nationally representative sample of public and private school students in grades 9–12 in the 50 U.S. states and the District of Columbia. Additional information about YRBS sampling, data collection, response rates, and processing is available in the overview report of this supplement (*17*). The prevalence estimates for report of unfair discipline at school and associations with multiple health risk behaviors and experiences for the overall study population and stratified by sex, race and ethnicity, grade, and sexual identity are available at https://nccd.cdc.gov/youthonline/App/Default.aspx. The full YRBS questionnaire, data sets, and documentation are available at https://www.cdc.gov/yrbs/index.html. Institutional review boards at CDC and ICF, the survey contractor, approved the protocol for YRBS. Data collection was conducted consistent with applicable Federal law and CDC policy.*

### Measures

The variable of interest was, “During the past 12 months, have you been unfairly disciplined at school?” with response options of yes or no. This measure was adapted from the Perceptions of Racism in Children and Youth scale, a scale demonstrated to be valid and reliable among youths of color who were ethnically and racially diverse (*18*). Research conducted among Black students demonstrated that students distinguish between disciplinary action that is discriminatory compared with discipline that is warranted (*5*). Health risk behaviors and experiences that were investigated in association with report of unfair discipline are grouped into three categories: 1) experiences at school, 2) mental health and suicidal thoughts and behaviors, and 3) health risk behaviors (Table 1). Demographic measures include sex (female or male), race and ethnicity (American Indian or Alaska Native [AI/AN], Asian, Black, Native Hawaiian or other Pacific Islander [NH/OPI], White, Hispanic or Latino [Hispanic], multiracial [selected >1 racial category] (persons of Hispanic origin might be of any race but are categorized as Hispanic; all racial groups are non-Hispanic), grade (9, 10, 11, or 12), and sexual identity (heterosexual or LGBQ+, which includes bisexual, gay, lesbian, questioning [I am not sure about my sexual identity/questioning], or describe identity in some other way [I describe my identity some other way]).

**TABLE 1:** Questions, response options, and analytic coding for included health risk behaviors and experiences among high school students — Youth Risk Behavior Survey, United States, 2023

Variable	Question	Response option	Analytic coding
**Experience at school**			
Bullied at school or electronically	Combined question: During the past 12 months, have you ever been bullied on school property? and During the past 12 months, have you ever been electronically bullied? (Count being bullied through texting, Instagram, Facebook, or other social media.)	Yes or no and yes or no	Yes (yes to either question) versus no (no to both questions)
Skipped school due to feeling unsafe	During the past 30 days, on how many days did you not go to school because you felt you would be unsafe at school or on your way to or from school?	0 days, 1 day, 2 or 3 days, 4 or 5 days, or ≥6 days	Yes (≥1 day) versus no (0 days)
Did not get mostly As or Bs	During the past 12 months, how would you describe your grades in school?	Mostly A's, mostly B's, mostly C's, mostly D's, mostly F's, none of these grades, or not sure	Do not get mostly A's and B's (mostly C's, mostly D's, mostly F's, none of these grades, or not sure) versus got mostly A's and B's (mostly A's or mostly B's)
**Mental health and suicidal thought or behavior**			
Poor mental health	During the past 30 days, how often was your mental health not good? (Poor mental health includes stress, anxiety, and depression.)	Never, rarely, sometimes, most of the time, or always	Yes (rarely, sometimes, most of the time, or always) versus no (never)
Persistent feelings of sadness or hopelessness	During the past 12 months, did you ever feel so sad or hopeless almost every time for two weeks or more in a row that you stopped doing some usual activities?	Yes or no	Yes versus no
Seriously considered attempting suicide	During the past 12 months, did you ever seriously consider attempting suicide?	Yes or no	Yes versus no
Attempted suicide	During the past 12 months, how many times did you actually attempt suicide?	0 times, 1 time, 2 or 3 times, 4 or 5 times, or ≥6 times	Yes (≥1 time) versus no (0 times)
**Health risk behavior**			
Carried a weapon at school	During the past 30 days, on how many days did you carry a weapon such as a gun, knife, or club on school property?	0 days, 1 day, 2 or 3 days, 4 or 5 days, or ≥6 days	Yes (≥1 day) versus no (0 days)
Ever prescription opioid misuse	During your life, how many times have you taken prescription pain medicine without a doctor's prescription or differently than how a doctor told you to use it?	0 times, 1 or 2 times, 3–9 times, 10–19 times, 20–39 times, or ≥40 times	Yes (≥1 time) versus no (0 times)
Poor sleep	On an average school night, how many hours of sleep do you get?	≤4 hours, 5 hours, 6 hours, 7 hours, 8 hours, 9 hours, or ≥10 hours	Yes (<8 hours) versus no (≥8 hours)

### Analysis

The prevalence of report of unfair discipline was estimated for all student respondents and stratified by race and ethnicity in combination with other demographic characteristics. Pairwise *t*-test analyses compared the prevalence of report of unfair discipline within a demographic characteristic. The prevalences of health behaviors and experiences were estimated among students who have and have not received unfair discipline. Logistic regression models were used to estimate the adjusted prevalence difference (aPD) and prevalence ratio (aPR) of each health behavior comparing student respondents who have and have not received unfair discipline. Differences were assessed on an absolute and relative scale. The prevalence estimates, aPDs, and aPRs are provided for all student respondents. In models for student respondents overall, aPDs and aPRs were adjusted for race and ethnicity, sex, and grade (operationalized as grades 9 and 10 versus grades 11 and 12), whereas models for racial and ethnic subgroups were adjusted only for sex and grade. Differences detected by *t*-test analyses were considered statistically significant at the p<0.05 level. Prevalence ratios were considered statistically significant if 95% CI did not cross the value of 1.0. Prevalence estimates with denominators <30 were considered statistically unreliable and therefore were suppressed (*17*), causing the results for NH/OPI students to not be presented for all analyses because of denominators <30. All prevalence estimates and measures of association were determined using Taylor series linearization. Prevalence difference and ratios were calculated using logistic regression with predicted marginals. All analyses were conducted using SAS-callable SUDAAN (version 11.0.3; RTI International) to account for the complex sampling design and weighting.

## Results

Overall, 19.3% of student respondents reported unfair discipline during the previous 12 months (Table 2). Black students (23.1%) had a higher prevalence of report of unfair discipline compared with Hispanic students (18.4%) and White students (18.1%). AI/AN students had the highest absolute point prevalence (32.0%) and NH/OPI the lowest (13.4%); however, because of wide confidence intervals in the response data for AI/AN and NH/OPI groups, these estimates were not different from the prevalence estimates of all other racial and ethnic subgroups. Male students and younger students (in grades 9 and 10) had the highest prevalence of report of unfair discipline compared with their respective peers. Students who identify their sexual identity in some other way (23.8%) had a higher prevalence of report of unfair discipline compared with questioning students (14.2%), and questioning students had a lower prevalence of report of unfair discipline compared with heterosexual students (18.9%).

**TABLE 2:** Prevalence of report of unfair discipline at school among high school students, by race and ethnicity and selected characteristics — Youth Risk Behavior Survey, United States, 2023*

Characteristic	AI/AN^†,§^ % (95% CI)	Asian^†,§^ % (95% CI)	Black or African American^†,§^ % (95% CI)	NH/OPI^†,§^ % (95% CI)	White^†,§^ % (95% CI)	Hispanic or Latino^†,§^ % (95% CI)	Multiracial^†,§^ % (95% CI)	Total % (95% CI)
Grade**								
9	28.7 (16.0–46.0)	25.2 (15.9–37.5)	28.6 (23.9–33.8)	—^††^	21.3 (17.5–25.7)	20.3 (16.8–24.3)	37.3 (27.9–47.8)	**23.6 (21.1–26.3)**
10	46.2 (24.1–70.0)	19.6 (13.0–28.5)	24.1 (19.1–29.9)	—	19.0 (16.2–22.1)	23.4 (19.8–27.4)	21.0 (13.0–32.2)	**21.1 (18.8–23.7)**
11	21.1 (8.0–45.0)	19.2 (11.7–29.9)	22.8 (16.7–30.3)	—	16.2 (13.4–19.4)	14.4 (11.7–17.7)	16.0 (8.1–29.2)	**16.7 (14.8–18.8)**
12	15.3 (7.0–30.3)	13.6 (7.8–22.8)	14.2 (7.9–24.4)	—	15.1 (12.6–17.9)	14.9 (10.5–20.8)	7.6 (4.6–12.5)	**14.4 (12.3–16.9)**
**Sex^§§^**								
Female	36.1 (19.2–57.3)	14.9 (9.6-22.5)	20.1 (15.7–25.3)	—	14.3 (12.3–16.5)	16.0 (14.2–18.0)	25.7 (19.5–33.0)	**16.4 (14.9–18.0)**
Male	26.1 (12.0–47.8)	22.2 (15.3–31.0)	26.2 (23.0–29.7)	—	21.0 (18.7–23.5)	20.7 (17.6–24.2)	19.8 (13.9–27.4)	**21.6 (19.9–23.4)**
**Sexual identity^¶¶^**								
LGBTQ+	45.8 (21.4–72.4)	19.0 (10.5–32.1)	20.0 (15.4–25.7)	—	17.3 (14.0–21.3)	15.1 (11.6–19.4)	32.6 (24.1–42.3)	**18.5 (15.9–21.4)**
Lesbian or gay	—	—	17.0 (8.7–30.4)	—	16.5 (10.1–25.6)	17.8 (9.2–31.4)	26.1 (13.8–43.6)	**18.3 (13.4–24.4)**
Bisexual	51.4 (26.7–75.5)	—	20.7 (14.6–28.5)	—	17.1 (12.9–22.3)	15.1 (9.4–23.5)	29.3 (19.9–40.9)	**18.3 (15.2–21.9)**
Questioning	12.1 (3.0–38.2)	—	12.6 (4.8–29.0)	—	12.4 (8.1–18.4)	16.4 (10.5–24.7)	17.9 (6.2–41.7)	**14.2 (10.2–19.3)**
Identify in some other way	81.7 (40.7–96.7)	—	28.2(14.7–47.2)	—	23.1 (15.6–32.6)	10.1 (3.5–25.8)	57.1 (39.0–73.6)	**23.8 (17.6–31.4)**
Heterosexual	26.6 (14.9–42.7)	18.3 (13.5–24.3)	23.8 (20.6–27.4)	16.2 (6.1–36.3)	18.0 (16.1–20.1)	18.6 (16.7–20.7)	16.7 (12.4–22.1)	**18.9 (17.6–20.2)**
**Total**	**32.0 (19.8–47.4)**	**19.0 (14.1–25.1)**	**23.1 (19.9–26.7)^¶^**	**13.4 (4.9–32.0)**	**18.1 (16.1–20.2)**	**18.4 (16.7–20.3)**	**22.4 (17.4–28.3)**	**19.3 (17.9–20.7)**

**Abbreviations:** AI/AN = American Indian or Alaska Native; NH/OPI = Native Hawaiian or other Pacific Islander.

* N = 20,103 respondents. The total number of students responding to each question varied. Data might be missing because 1) the question did not appear in that student’s questionnaire, 2) the student did not answer the question, or 3) the response was set to missing because of an out-of-range response or logical inconsistency. Percentages in each category are calculated on the known data.

^†^ The total number of respondents varied by race and ethnicity category, with the following unweighted number of students responding to the discipline question: AI/AN = 839; Asian = 402; Black or African American = 1,242; NH/OPI =53; White = 4,568; Hispanic or Latino = 2,289; and multiracial = 1,078. Data might be missing because 1) the question did not appear in that student’s questionnaire, 2) the student did not answer the question, or 3) the response was set to missing because of an out-of-range response or logical inconsistency. Percentages in each category are calculated on the known data.

^§^ Persons of Hispanic or Latino origin might be of any race but are categorized as Hispanic; all racial groups are non-Hispanic.

^¶^ Significantly different from Hispanic or Latino and White students, based on *t*-test analysis with Taylor series linearization (p<0.05).

** Among students overall, those in grades 9 and 10 significantly differed from students in grades 11 and 12. Among AI/AN students, students in grade 10 significantly differed from students in grade 12. Among Asian students, students in grade 9 significantly differed from students in grade 12. Among Black or African American students, students in grades 9 and 10 significantly differed from students in grade 12. Among Hispanic or Latino students, students in grade 9 significantly differed from students in grade 11, and students in grade 10 significantly differed from students in grades 11 and 12. Among multiracial students, students in grade 9 significantly differed from students in grades 10, 11, and 12, and students in grade 10 significantly differed from students in grade 12. Among White students, students in grade 9 significantly differed from students in grades 11 and 12, and students in grade 10 significantly differed from students in grade 12. Significance based on *t*-test analysis with Taylor series linearization (p<0.05).

^††^ Dashes indicate where prevalence estimates were suppressed because n<30.

^§§^ Female students significantly differed from male students among students overall, Black or African American students, Hispanic or Latino students, and White students, based on *t*-test analyses with Taylor series linearization (p<0.05)

^¶¶^ Among students overall, questioning students significantly differed from heterosexual students and students who identify in some other way. Among AI/AN students, students who identify in some other way significantly differed from heterosexual and questioning students, and questioning students significantly differed from bisexual students. Among Black or African American students, students who were questioning significantly differed from heterosexual students. Among multiracial students, LGBQ+ students significantly differed from heterosexual students, bisexual students significantly differed from heterosexual students, and students who identify in some other way significantly differed from heterosexual, lesbian or gay, bisexual, and questioning students. Among White students, questioning students significantly differed from heterosexual students. Significance based on *t*-test analysis with Taylor series linearization (p<0.05).

In all racial and ethnic subgroups, students in either grade 9 or 10 had the highest prevalence of report of unfair discipline. Male students had a higher prevalence of report of unfair discipline than female students among Black (26.2% versus 20.1%), Hispanic (20.7% versus 16.0%), and White (21.0% versus 14.3%) students. Questioning students had a lower prevalence of report of unfair discipline compared with heterosexual students among Black (12.6% versus 23.8%) and White (12.4% versus 18.0%) students. Many differences by sexual identity were noted among AI/AN and multiracial students, and students who identified in some other way most differed from the remaining sexual identity categories. Among AI/AN students, students who identified in some other way (81.7%) had a higher report of unfair discipline compared with questioning (12.1%) and heterosexual (26.6%) students. Among multiracial students, students who identified in some other way (57.1%) had a higher prevalence of report of unfair discipline compared with bisexual (29.3%), lesbian or gay (26.1%), questioning (17.9%), and heterosexual (16.7%) students. In addition, among multiracial students, LGBTQ+ students (32.6%) had a higher report of unfair discipline compared with heterosexual (16.7%) students.

Among students overall, report of unfair discipline was associated with a higher prevalence of every health risk behavior and experience on both an absolute and relative scale (Table 3). For example, associations encompassed experiences at school, including having been bullied at school or electronically, skipped school due to feeling unsafe, or did not get mostly As and Bs; health risk behaviors, including carried a weapon at school, ever prescription opioid misuse, or poor sleep; mental health problems, including poor mental health and persistent feelings of sadness or hopelessness; and suicide risk, including seriously considered attempting suicide and attempted suicide.

**TABLE 3:** Prevalences, adjusted prevalence differences, and adjusted prevalence ratios for high school students that did and did not report receipt of unfair discipline at school, by selected health risk behaviors and experiences — Youth Risk Behavior Survey, United States, 2023*

Health risk behavior or experience^†^	Students that reported unfair discipline % (95% CI)	Students that did not report unfair discipline % (95% CI)	aPD^§^	aPR^§^	95% CI
**Experience at school**					
Bullied at school or electronically	41.6 (37.8–45.5)	20.9 (19.4–22.6)	21.7^¶^	2.05**	1.87–2.25
Skipped school due to feeling unsafe	21.8 (17.7–26.5)	11.2 (8.4–14.9)	11.2^¶^	2.00**	1.63–2.47
Did not get mostly As and Bs	37.3 (34.1–40.6)	25.4 (22.1–29.0)	10.6^¶^	1.42**	1.24–1.62
**Mental health and suicidal thought or behavior**					
Poor mental health	38.0 (34.5–41.7)	27.2 (25.1–29.3)	13.2^¶^	1.50**	1.34–1.66
Persistent feelings of sadness or hopelessness	53.1 (50.3–55.8)	37.5 (35.0–40.0)	18.1^¶^	1.49**	1.40–1.58
Seriously considered attempting suicide	29.1 (26.8–31.5)	17.5 (15.9–19.3)	12.5^¶^	1.73**	1.54–1.93
Attempted suicide	15.3 (13.2–17.6)	7.3 (6.4–8.4)	8.1^¶^	2.12**	1.80–2.48
**Health risk behavior**					
Carried a weapon at school	8.3 (6.1–11.3)	3.1 (2.1–4.5)	4.7^¶^	2.52**	1.90–3.35
Ever prescription opioid misuse	17.8 (15.8–20.0)	9.1 (8.1–10.2	8.5^¶^	1.95**	1.69–2.24
Poor sleep	81.2 (77.6–84.3)	75.5 (73.5–77.4)	7.0^¶^	1.09**	1.06–1.13

**Abbreviations:** aPD = adjusted prevalence difference; aPR = adjusted prevalence ratio.

* N = 20,103 respondents. The total number of students answering each question varied. Data might be missing because 1) the question did not appear in that student’s questionnaire, 2) the student did not answer the question, or 3) the response was set to missing because of an out-of-range response or logical inconsistency. Percentages in each category are calculated on the known data.

^†^ Refer to Table 1 for variable definitions.

^§^ aPDs and aPRs calculated using logistic regression models with predicted marginal proportions adjusted for race and ethnicity, grade, and sex.

^¶^ Statistically significant based on a pairwise difference from the aPD logistic regression model with predicted marginal proportions (p<0.05).

** Statistically significant; 95% CIs do not cross the value of 1.0.

Comparisons by race and ethnicity were made to facilitate the examination of the association between report of unfair discipline and health and well-being within each racial and ethnic subgroup. Most behaviors among Black, Hispanic, and multiracial students and all behaviors among White students were associated with report of unfair discipline on an absolute and relative scale (Table 4). In addition, all racial and ethnic subgroups had at least one association on the absolute and relative scales between report of unfair discipline and mental health and suicidal thoughts and behaviors. Among all racial and ethnic subgroups, approximately half of students who received unfair discipline had persistent feelings of sadness or hopelessness (ranging from 48.4% among Asian students to 77.6% among AI/AN students), approximately one fourth to one third seriously considered attempting suicide (ranging from 22.4% among Asian students to 36.8% among AI/AN students), and more than one in 10 students attempted suicide (ranging from 12.4% among Black students to 20.7% among multiracial students).

**TABLE 4:** Prevalences and 95% CIs, adjusted prevalence differences, and adjusted prevalence ratios for high school students that did and did not report receipt of unfair discipline at school, by race and ethnicity and selected health risk behaviors and experiences — Youth Risk Behavior Survey, United States, 2023*

Race or ethnicity^†^	Health risk behavior or experience^§^	Student reporting unfair discipline^¶^ % (95% CI)	Student not reporting unfair discipline^¶^ % (95% CI)	aPD**	aPR**	95% CI
American Indian or Alaska Native	**Experience at school**					
	Bullied at school or electronically	33.2 (14.8–58.9)	17.6 (10.7–27.4)	15.9	1.91	0.86–4.22
	Skipped school due to feeling unsafe	20.4 (6.7–47.8)	11.7 (5.8–22.2)	7.0	1.58	0.48–5.18
	Did not get mostly As and Bs	30.4 (14.0–54.1)	40.4 (26.2–56.5)	−3.6	0.91	0.52–1.59
	**Mental health and suicidal thought or behavior**					
	Poor mental health	26.4 (10.1–53.3)	34.0 (21.6–49.0)	−12.2	0.66	0.25–1.70
	Felt persistently sad or hopeless	77.6 (55.4–90.7)	43.9 31.2–57.6)	29.5^††^	1.65^§§^	1.16–2.34
	Seriously considered attempting suicide	36.8 (15.3–65.1)	13.3 (8.8–19.8)	23.1	2.72^§§^	1.17–6.32
	Attempted suicide	14.1 (5.7–31.0)	10.2 (6.3–16.2)	2.7	1.25	0.44–3.58
	**Health risk behavior**					
	Carried a weapon at school	1.1 (0.4–3.2)	1.4 (0.8–2.7)	—^¶¶^	0.98	0.35–2.75
	Ever prescription opioid misuse	11.2 (4.5–25.4)	12.8 (6.0–25.2)	−3.0	0.78	0.20–2.96
	Poor sleep	78.7 (46.2–94.1)	75.0 (57.7–86.9)	8.4	1.11	0.82–1.52
Asian	**Experience at school**					
	Bullied at school or electronically	31.0 (18.5–47.1)	17.1 (12.0–23.6)	13.7^††^	1.79^§§^	1.17–2.76
	Skipped school due to feeling unsafe	22.3 (10.8–40.4)	7.9 (3.9–15.2)	12.1^††^	2.49^§§^	1.58–3.90
	Did not get mostly As and Bs	17.3 (9.8–28.6)	14.4 (7.9–24.8)	1.1	1.07	0.58–2.00
	**Mental health and suicidal thought or behavior**					
	Poor mental health	32.9 (19.2–50.2)	19.9 (14.3–26.9)	15.6	1.80^§§^	1.08–3.01
	Felt persistently sad or hopeless	48.4 (35.2–61.9)	25.4 (19.5–32.3)	24.1^††^	1.96^§§^	1.44–2.66
	Seriously considered attempting suicide	22.4 (14.8–32.4)	9.7 (6.5–14.6)	14.1^††^	2.48^§§^	1.42–4.34
	Attempted suicide	17.5 (10.1–28.8)	6.4 (3.2–12.6)	11.4^††^	2.81^§§^	1.63–4.84
	**Health risk behavior**					
	Carried a weapon at school	4.4 (1.0–16.8)	2.2 (0.9–5.0)	1.5	1.06	0.38–2.97
	Ever prescription opioid misuse	17.5 (8.4–33.1)	8.2 (3.6–17.9)	9.2	2.11	0.64–6.99
	Poor sleep	79.6 (62.4–90.1)	83.0 (77.7–87.2)	−3.4	0.96	0.80–1.14
Black or African American	**Experience at school**					
	Bullied at school or electronically	29.8 (23.1–37.6)	13.6 (11.0–16.7)	16.3^††^	2.19^§§^	1.52–3.14
	Skipped school due to feeling unsafe	23.2 (15.4–33.4)	13.9 (9.0–20.8)	9.7	1.70	0.91–3.19
	Did not get mostly As and Bs	40.0 (35.6–44.5)	29.8 (24.5–35.8)	9.2^††^	1.31^§§^	1.05–1.63
	**Mental health and suicidal thought or behavior**					
	Poor mental health	38.4 (32.7–44.4)	24.2 (20.1–28.9)	16.8^††^	1.71^§§^	1.36–2.14
	Felt persistently sad or hopeless	52.1 (46.6–57.7)	36.4 (33.2–39.7)	19.1^††^	1.53^§§^	1.32–1.78
	Seriously considered attempting suicide	23.3 (19.9–27.1)	16.9 (13.3–21.2)	8.2^††^	1.49^§§^	1.13–1.97
	Attempted suicide	12.4 (9.4–16.1)	7.5 (5.7–9.8)	5.3^††^	1.71^§§^	1.15–2.54
	**Health risk behavior**					
	Carried a weapon at school	5.0 (2.6–9.4)	1.9 (1.2–3.1)	2.8	2.40^§§^	1.14–5.05
	Ever prescription opioid misuse	18.2 (14.4–22.7)	9.4 (7.1–12.2)	9.0^††^	1.96^§§^	1.45–2.64
	Poor sleep	82.2 (67.0–91.3)	78.8 (72.4–84.0)	5.2	1.07	0.93–1.22
White	**Experience at school**					
	Bullied at school or electronically	48.1 (44.0–52.1)	26.0 (23.9–28.1)	22.9^††^	1.89^§§^	1.66–2.15
	Skipped school due to feeling unsafe	20.8 (16.7–25.5)	9.3 (7.1–12.1)	12.1^††^	2.32^§§^	1.90–2.84
	Did not get mostly As and Bs	34.0 (29.9–38.3)	18.4 (15.5–21.8)	13.7^††^	1.74^§§^	1.49–2.03
	**Mental health and suicidal thought or behavior**					
	Poor mental health	40.2 (35.9–44.7)	29.5 (26.6–32.7)	13.3^††^	1.46^§§^	1.29–1.65
	Felt persistently sad or hopeless	53.8 (49.6–58.0)	36.9 (33.6–40.2)	20.1^††^	1.56^§§^	1.42–1.70
	Seriously considered attempting suicide	32.8 (29.7–36.0)	19.3 (17.2–21.5)	14.9^††^	1.78^§§^	1.59–2.00
	Attempted suicide	14.1 (10.9–18.1)	6.7 (5.3–8.5)	7.9^††^	2.18^§§^	1.74–2.74
	**Health risk behavior**					
	Carried a weapon at school	9.5 (5.8–15.4)	3.8 (2.2–6.3)	5.2^††^	2.36^§§^	1.73–3.23
	Ever prescription opioid misuse	16.3 (13.2–19.9)	8.1 (6.6–10.0)	8.6^††^	2.07^§§^	1.55–2.77
	Poor sleep	81.9 (76.9–86.0)	74.2 (71.6–76.5)	8.6^††^	1.12^§§^	1.06–1.18
Hispanic or Latino	**Experience at school**					
	Bullied at school or electronically	36.4 (28.3–45.4)	17.6 (15.4–20.1)	19.5^††^	2.13^§§^	1.72–2.64
	Skipped school due to feeling unsafe	23.7 (17.0–32.1)	14.0 (9.9–19.4)	10.7^††^	1.78^§§^	1.40–2.26
	Did not get mostly As and Bs	43.2 (37.0–49.7)	36.2 (30.9–41.7)	6.8	1.19	0.97–1.45
	**Mental health and suicidal thought or behavior**					
	Poor mental health	33.0 (25.5–41.6)	26.3 (23.8–28.9)	8.6^††^	1.33^§§^	1.04–1.71
	Felt persistently sad or hopeless	50.9 (45.4–56.4)	40.9 (37.1–44.8)	12.2^††^	1.30^§§^	1.18–1.44
	Seriously considered attempting suicide	25.2 (19.6–31.9)	15.8 (13.9–18.0)	10.6^††^	1.68^§§^	1.34–2.12
	Attempted suicide	17.1 (13.3–21.8)	7.6 (6.6–8.7)	9.6^††^	2.30^§§^	1.71–3.11
	**Health risk behavior**					
	Carried a weapon at school	8.3 (5.2–12.9)	2.9 (1.9–4.2)	4.8^††^	2.71^§§^	1.47–5.01
	Ever prescription opioid misuse	19.1 (15.4–23.4)	10.8 (9.3–12.4)	8.0^††^	1.76^§§^	1.28–2.42
	Poor sleep	80.0 (75.2–84.0)	74.5 (70.3–78.3)	7.3^††^	1.10^§§^	1.01–1.19
Multiracial	**Experience at school**					
	Bullied at school or electronically	56.6 (46.3–66.3)	16.0 (11.6–21.7)	37.9^††^	3.31^§§^	2.34–4.69
	Skipped school due to feeling unsafe	20.8 (12.2–33.2)	8.6 (4.9–14.5)	11.2^††^	2.29^§§^	1.21–4.34
	Did not get mostly As and Bs	38.8 (29.9–48.6)	24.0 (16.6–33.3)	13.8^††^	1.58^§§^	1.01–2.50
	**Mental health and suicidal thought or behavior**					
	Poor mental health	47.2 (36.2–58.6)	24.4 (19.3–30.3)	22.1^††^	1.91^§§^	1.41–2.60
	Felt persistently sad or hopeless	61.4 (53.4–68.8)	37.5 (30.6–44.9)	22.6^††^	1.59^§§^	1.32–1.92
	Seriously considered attempting suicide	32.6 (21.4–46.3)	18.8 (13.1–26.4)	12.1^††^	1.64^§§^	1.02–2.66
	Attempted suicide	20.7 (12.9–31.7)	10.3 (7.2–14.6)	8.4	1.79^§§^	1.01–3.17
	**Health risk behavior**					
	Carried a weapon at school	8.0 (4.4–14.2)	2.9 (1.4–5.8)	5.9	3.03	0.94–9.77
	Ever prescription opioid misuse	18.4 (13.1–25.3)	8.1 (5.5–11.7)	10.9^††^	2.35^§§^	1.39–3.96
	Poor sleep	82.6 (73.2–89.2)	81.2 (76.4–85.1)	4.0	1.05	0.96–1.14

**Abbreviations:** aPD = adjusted prevalence difference; aPR = adjusted prevalence ratio.

* The total number of respondents varied by race and ethnicity category, with the following unweighted number of students responding to the discipline question: AI/AN = 839; Asian = 402; Black or African American = 1,242; White = 4,568; Hispanic or Latino = 2,289; and multiracial = 1,078. Data might be missing because 1) the question did not appear in that student’s questionnaire, 2) the student did not answer the question, or 3) the response was set to missing because of an out-of-range response or logical inconsistency. Percentages in each category are calculated on the known data.

^†^ Persons of Hispanic or Latino origin might be of any race but are categorized as Hispanic; all racial groups are non-Hispanic.

^§^ Refer to Table 1 for variable definitions.

^¶^ Prevalence estimates and 95% CIs are not adjusted.

** aPD and aPR calculated using logistic regression models with predicted marginal proportions adjusted for grade and sex.

^††^ Statistically significant based on a pairwise difference from the aPD logistic regression model with predicted marginal proportions (p<0.05).

^§§^ Statistically significant; 95% CIs do not cross the value of 1.0.

^¶¶^ aPD = 0.0.

## Discussion

Unfair discipline at school was demonstrated to be associated with a higher prevalence of every health risk behavior and experience examined, including being bullied at school or electronically, skipping school due to feeling unsafe, not getting mostly As and Bs, poor mental health, persistent feelings of sadness or hopelessness, seriously considering attempting suicide, attempting suicide, carrying a weapon at school, poor sleep, and prescription opioid misuse. These findings demonstrate that school discipline is an urgent public health problem. The importance of these findings is underscored by this being the first report to investigate the association between school discipline and health and well-being using a nationally representative survey sample of U.S. high school students.

Among students who reported receiving unfair discipline at school, more than half reported persistent feelings of sadness or hopelessness, approximately one fourth to one third seriously considered attempting suicide, and more than one in 10 students attempted suicide. These findings are consistent with other research indicating that being suspended from school is associated with poor mental health and death by suicide (*10*). Students who reported receiving unfair discipline were also found to be more likely to report being bullied at school or electronically and skipping school due to feeling unsafe compared with students who did not report unfair discipline. Previous studies have found a relation between unfair discipline and negative experiences at school or avoiding school among students who experience discrimination. For example, LGBTQ+ students who are bullied also report receiving discipline related to their bullying victimization experience (*19*). Among Black students, the receipt of unfair discipline is a risk factor for skipping school or even changing school districts (*5*). Considering the dramatic increase in chronic absenteeism in recent years (https://www2.ed.gov/datastory/chronicabsenteeism.html), these findings suggest that school officials might need to consider alternatives to current approaches to discipline because by skipping school, certain students might be trying to avoid the experience of receiving discipline (*5*). Creating school environments that are inclusive and fair, while also maintaining safety, is a Federal strategy to ensure that all students succeed in school (*16*).

Among students overall, 19.3% of students reported receiving unfair discipline at school, and Black students (23.1%) were the only racial or ethnic group to have a significantly higher prevalence than other racial or ethnic groups (different from Hispanic and White students). This disproportionate report of unfair discipline is also demonstrated by data on disproportionate disciplinary practices at the school level, collected by the Office for Civil Rights within the U.S. Department of Education, which indicate that Black students in K–12 public schools are disciplined at a rate that is higher than any other racial or ethnic group (*15*). These racial disparities cannot be explained by differences in socioeconomic status (*3*,*13*), behavior (*3*,*4*), or academic performance (*14*). The disproportionate discipline that Black students receive is rooted in racially discriminatory policies and practices (i.e., structural racism) (*20*), which began with school desegregation and has continued to the present day (*12*). Black students also disproportionately experience consequences from receiving school discipline compared with White students, such as lower academic achievement (*4*,*7*), chronic absenteeism and dropping out (*4*,*5*), and arrest (*4*,*6*). The field of public health might benefit from using this evidence base to analyze data for action to create strategies to help the one in five students, overall, who report receiving unfair discipline.

A salient finding from this report is that 81.7% of AI/AN students and 57.1% of multiracial students who identify in some other way (as a sexual identity) reported receiving unfair discipline at school. In addition, one third of multiracial students who identify as LGBQ+ reported unfair discipline. The Office for Civil Rights does not collect information on students’ sexual and gender identity, so this finding represents a new data point in nationally representative data that document the experience of LGBQ+ students, specifically LGBQ+ students of color (AI/AN and multiracial students), and students who face discrimination because of both their racial and ethnic and sexual identities. Relatively little research has been conducted on students with both AI/AN or multiracial and LGBTQ+ identities, particularly in the area of unfair discipline; however, previous studies have found that school disciplinary action is applied disproportionately to LGBTQ+ students (*3*). The U.S. Department of Education also recognizes youths of color and LGBTQ+ students as groups who receive disproportionate discipline (*16*).

Schools play a vital role in creating safe and supportive environments that promote the well-being of all students; however, this report contributes to the literature that demonstrates the negative experiences associated with school discipline. Research demonstrates that racial disparities in discipline might be in part attributable to teacher and school administrator perceptions and attitudes (*3*,*14*), which might include racial bias and other forms of discrimination. Offering school-based supports (e.g., implementing curricula inclusive of LGBTQ+ topics and establishing affinity groups, such as genders and sexualities alliances) has been found to reduce health risk behaviors and experiences (*21*), and extending these practices to include professional development on cultural bias and anti-racist practices, as well as creating ethnic or cultural affinity clubs, might foster a safe and supportive environment that promotes equity. For example, Boston Public Schools has developed the Equity Impact Analysis Tool (https://www.bostonpublicschools.org/cms/lib/MA01906464/Centricity/Domain/162/BPS%20Racial%20Equity%20Impact%20Tool%20in%20Word.pdf) to help school and district leaders determine whether existing and proposed policies, budget allocations, programs, professional development, and instructional practices are likely to close opportunity gaps for students with identities that have been marginalized.

These strategies are aligned with the U.S. Department of Education’s Guiding Principles for Creating Safe, Inclusive, Supportive, and Fair School Climates (https://www2.ed.gov/policy/gen/guid/school-discipline/guiding-principles.pdf), which provides five guiding principles schools can follow to apply discipline fairly. Examples include creating an inclusive and welcoming environment for all students, hiring and maintaining a diverse school workforce, and involving the entire school community (students, parents, teachers, school staff members, and school leaders) in crafting fair disciplinary practices and tracking their fair implementation. Because of the finding that report of unfair discipline is associated with poor mental health and suicidal thoughts and behaviors, the importance of using data for action to nurture positive mental health and well-being for students who report unfair discipline cannot be overstated. To help address these findings, school leaders might choose to implement the six strategies from Promoting Mental Health and Well-Being in Schools: An Action Guide for School and District Leaders (https://www.cdc.gov/healthyyouth/mental-health-action-guide/pdf/DASH_MH_Action_Guide_508.pdf), which outlines how focusing on the diverse needs of students, promoting health equity, and providing opportunities for school staff members to receive training and services on mental health (among other strategies and approaches) can foster student mental health and well-being. The educational and public health guiding documents complement one another, providing school communities with a robust roadmap to prevent using discipline inequitably and address the health needs of students who have experienced discipline.

## Limitations

General limitations for the YRBS are available in the overview report of this supplement (*17*). The findings in this report are subject to at least three additional limitations. First, because the data are cross-sectional, the variables associated with report of unfair discipline might be thought of as outcomes but they might in fact be co-occurring or serve as the reason for report of unfair discipline. Second, report of unfair discipline is self-reported experience; however, qualitative and quantitative research demonstrates that students are able to identify when discipline is unfair (*5*,*13*,*18*). Finally, students whose unfair discipline experience included expulsion might be underrepresented in this survey.

## Future Directions

Future studies could use local school district YRBS data to examine associations between disciplinary practices and health. District administrators could use the YRBS data, along with other data available within their district, to triangulate findings that reveal more about the relation between discrimination, unfair discipline, and health. By centering the research conducted with Black students about their experiences with inequitable discipline, public health practitioners can learn from the established evidence to understand the experiences of other students who experience inequitable or unfair discipline, especially students who have identities that face bias and discrimination. For example, the finding that most AI/AN and multiracial students who identify their sexuality in some other way report receiving unfair discipline warrants additional exploration. The U.S. Department of Education also has described widespread and consistent disparities in school discipline between students with and without disabilities, and future research should examine the relation between discipline and health among students with disabilities. This report calls for public health practitioners, school administrators, families, youths, and community partners to reassess their district’s current discipline-related policies and procedures, to recognize their association with health risk behaviors and experiences among students, and to create interventions that equitably promote safe, supportive, and inclusive school environments for student health.

## Conclusion

Findings from this report provide considerable evidence that student report of unfair discipline at school is associated with poor mental health, suicidal thoughts and behaviors, and experiences of violence, in addition to concerning behavior such as prescription opioid misuse and skipping school due to feeling unsafe. These data are the first to present rigorous evidence from a nationally representative sample of U.S. high school students that links student report of unfair school discipline to health risk behaviors and experiences, foregrounding the current use of school discipline as an urgent public health concern. With these findings, public health and education practitioners can create interventions that equitably promote safe, supportive, and inclusive school environments for student health.

## Footnotes

* 45 C.F.R. part 46.114; 21 C.F.R. part 56.114.

## References

[1] U.S. Department of Education. School climate and student discipline resources. Washington, DC: US Department of Education; 2022. https://www2.ed.gov/policy/gen/guid/school-discipline/index.html

[2] Office for Civil Rights, US Department of Education, Civil Rights Division, U.S. Department of Justice. Resource on confronting racial discrimination in student discipline. Washington, DC: US Department of Education and US Department of Justice; 2023. https://www2.ed.gov/about/offices/list/ocr/docs/tvi-student-discipline-resource-202305.pdf

[3] Welsh RO, Little S. The school discipline dilemma: a comprehensive review of disparities and alternative approaches. Rev Educ Res 2018;88:752–94. 10.3102/0034654318791582

[4] Welsh RO, Little S. Caste and control in schools: a systematic review of the pathways, rates and correlates of exclusion due to school discipline. Child Youth Serv Rev 2018;94:315–39. 10.1016/j.childyouth.2018.09.031

[5] Bell C. Suspended: punishment, violence, and the failure of school safety. 1st ed. Baltimore, MD: Johns Hopkins University Press; 2021.

[6] Fisher BW, Widdowson AO. Racial and ethnic differences in the consequences of school suspension for arrest. Criminology 2023;61:622–53. 10.1111/1745-9125.12344

[7] Morris EW, Perry BL. The punishment gap: school suspension and racial disparities in achievement. Soc Probl 2016;63:68–86. 10.1093/socpro/spv026

[8] Lamont JH, Devore CD, Allison M, ; Council on School Health. Out-of-school suspension and expulsion. Pediatrics 2013;131:e1000–7. 10.1542/peds.2012-3932

[9] American Psychological Association Zero Tolerance Task Force. Are zero tolerance policies effective in the schools?: an evidentiary review and recommendations. Am Psychol 2008;63:852–62. 10.1037/0003-066X.63.9.852 19086747

[10] Duarte CD, Moses C, Brown M, Punitive school discipline as a mechanism of structural marginalization with implications for health inequity: a systematic review of quantitative studies in the health and social sciences literature. Ann N Y Acad Sci 2023;1519:129–52. 10.1111/nyas.14922 36385456 PMC10929984

[11] US Department of Education. School climate and student discipline resources: know the data. Washington, DC: US Department of Education; 2022. https://www2.ed.gov/policy/gen/guid/school-discipline/data.html

[12] Skiba R, White A. Ever since Little Rock: the history of disciplinary disparities in America’s schools. In: Gage N, Rapa LJ, Whitford DK, Katsiyannis A, eds. Disproportionality and social justice in education. Cham, Switzerland: Springer International Publishing; 2022:3–33.

[13] Wallace JM Jr, Goodkind S, Wallace CM, Bachman JG. Racial, ethnic, and gender differences in school discipline among U.S. high school students: 1991–2005. Negro Educ Rev 2008;59:47–62. 19430541 PMC2678799

[14] Rocque M, Paternoster R. Understanding the antecedents of the “school-to-jail” link: the relationship between race and school discipline. J Crim Law Criminol 2011;101:633–65.

[15] Office for Civil Rights. 2020–21 Civil rights data collection: student discipline and school climate in U.S. public schools. Washington, DC: US Department of Education, Office for Civil Rights; 2023.

[16] U.S. Department of Education. Guiding principles for creating safe, inclusive, supportive, and fair school climates. Washington, DC: US Department of Education; 2023. https://www2.ed.gov/policy/gen/guid/school-discipline/guiding-principles.pdf

[17] Brener ND, Mpofu JJ, Krause KH, Overview and methods for the Youth Risk Behavior Surveillance System—United States, 2023. In: Youth Risk Behavior Surveillance—United States, 2023. MMWR Suppl 2024;73(No. Suppl-4):1–12. 10.15585/mmwr.su7304a1 PMC11559678 39378301

[18] Pachter LM, Szalacha LA, Bernstein BA, García Coll C. Perceptions of Racism in Children and Youth (PRaCY): properties of a self-report instrument for research on children’s health and development. Ethn Health 2010;15:33–46. 10.1080/13557850903383196 20013438 PMC2891186

[19] Horn SS, Schriber SE. Bullied and punished: exploring the links between bullying and discipline for sexual and gender minority youth. J Res Adolesc 2020;30:735–52. 10.1111/jora.12556 32175649

[20] Crutchfield J, Phillippo KL, Frey A. Structural racism in schools: a view through the lens of the national school social work practice model. Child Schools 2020;42:187–93. 10.1093/cs/cdaa015

[21] Kaczkowski W, Li J, Cooper AC, Robin L. examining the relationship between LGBTQ-supportive school health policies and practices and psychosocial health outcomes of lesbian, gay, bisexual, and heterosexual students. LGBT Health 2022;9:43–53. 10.1089/lgbt.2021.0133 34935516 PMC9003132

